# The Anti-inflammatory Mediator Resolvin E1 Protects Mice Against Lipopolysaccharide-Induced Heart Injury

**DOI:** 10.3389/fphar.2020.00203

**Published:** 2020-03-18

**Authors:** Jishou Zhang, Menglong Wang, Jing Ye, Jianfang Liu, Yao Xu, Zhen Wang, Di Ye, Mengmeng Zhao, Jun Wan

**Affiliations:** ^1^Department of Cardiology, Renmin Hospital of Wuhan University, Wuhan, China; ^2^Cardiovascular Research Institute, Wuhan University, Wuhan, China; ^3^Hubei Key Laboratory of Cardiology, Wuhan, China

**Keywords:** sepsis-induced cardiomyopathy, resolvin E1, inflammation, mitogen-activated protein kinase, macrophage polarization, apoptosis

## Abstract

**Background:**

Sepsis-induced cardiomyopathy (*SIC*) is a common severe complication of sepsis that contributes to mortality. *SIC* is closely associated with excessive inflammatory responses, failed inflammation resolution, and apoptotic damage. Resolvin E1 (RvE1), an omega-3 polyunsaturated fatty acid (PUFA)-derived metabolite, has been reported to exert anti-inflammatory or proresolving activity in multiple animal models of inflammatory disease. However, the therapeutic potential of RvE1 in *SIC* remains undetermined, which was, therefore, the aim of the present study.

**Methods:**

C57BL/6J mice were randomly divided into three groups: control, lipopolysaccharide (LPS), and LPS + RvE1. Echocardiography, Western blotting (WB), quantitative real-time (QRT)-PCR, histological analyses, and flow cytometry were used to evaluate cardiac function, myocardial inflammation, and the underlying mechanisms.

**Results:**

The RvE1-injected group showed improved left ventricular (LV) function and reduced serum lactate dehydrogenase (LDH) and creatine kinase myocardial bound (CK-MB) levels. Compared to LPS treatment alone, RvE1 treatment inhibited the infiltration of neutrophils and macrophages into the heart and spleen and suppressed the secretion of pro-inflammatory cytokines, including interleukin (IL)-1β, IL-6, and monocyte chemoattractant protein (MCP)-1, in the heart. We also observed that the activation of the mitogen-activated protein kinase (MAPK) and nuclear factor (NF)-κB signaling pathways was blocked by RvE1 treatment, and this inhibition contributed to the improvement in the inflammatory response induced by LPS. RvE1 inhibited LPS-induced M1 macrophage polarization and promoted macrophage polarization toward the M2-like phenotype in both the heart and spleen. In addition, LPS administration dysregulated cyclooxygenase (COX) and lipoxygenase (LOX) in the heart, which were rectified by RvE1 treatment. RvE1 also reduced myocardial apoptosis rate in response to LPS-induced heart injury.

**Conclusion:**

RvE1 protects the heart against *SIC* possibly through the inhibition of the MAPK and NF-κB inflammatory signaling pathways, modulation of macrophage polarization, and reduction in myocardial apoptosis. RvE1 may be a novel lipid mediator for the treatment of *SIC*.

## Introduction

Sepsis is defined as life-threatening organ dysfunction and is caused by a dysregulated host response to infection. LPS from the bacterial cell wall is frequently used for the induction of sepsis (Xianchu et al., 2018). Sepsis-induced cardiomyopathy is a common complication of severe sepsis and is closely associated with the prognosis of patients. Evidence has suggested that myocardial injury caused by sepsis has characteristics of an excessive inflammatory response, failed resolution of inflammation, and apoptotic damage, which may ultimately bring about qualitative and quantitative myocardial alterations (Sharma, 2007; Deutschman and Tracey, 2014; Kong et al., 2017). Therefore, it is imperative that a novel strategy to protect patients against sepsis-induced myocardial injury *via* the inhibition of inflammation, promotion of inflammation resolution, or inhibition of cardiac myocyte apoptosis is found. In fact, promoting the switch from the inflammation phase to the resolution phase is a significant method to prevent heart injury (Cheng et al., 2017) and has become a hotpot about popular approach for regulating inflammation.

Anti-inflammatory lipid chemokines, including lipoxins, resolvins, protectins, and maresins, have shown remarkable beneficial effects in several animal models of disease, including atherosclerosis, diabetes, and obesity (Serhan, 2017). Resolvin E1 (RvE1) is one such chemokine. RvE1 is biosynthesized from EPA, which is metabolized to produce 18R-hydroxy-5Z,8Z,11Z,14Z,16E-EPA (18R-HEPE) *via* aspirin-acetylated COX-2 in endothelial cells or *via* a COX-independent pathway involving cytochrome P450 and is subsequently transformed by 5-LOX in neutrophils (Arita et al., 2005). The proresolving activity of RvE1 is mediated by the receptor ERV1/ChemR23, which is a G-protein-coupled receptor (Arita et al., 2005; Pirault and Back, 2018). The overexpression of ERV1/ChemR23 enhances the protective impact of RvE1 in several animal models of inflammation (Gao et al., 2013; Herrera et al., 2015; Sima et al., 2017). In addition, RvE1, showing as an antagonist, interacts with the BLT1 and ultimately attenuates LB4-dependent inflammation (Arita et al., 2007).

Previous studies indicated that RvE1 has remarkable anti-inflammatory and proresolution effects in many diseases, such as keratitis, periodontitis, allergic asthma, bacterial pneumonia, and acute lung injury (Seki et al., 2010; Flesher et al., 2014; Lee et al., 2015, 2016). Recent trials have shown that in the cardiovascular system, RvE1 prevents vascular inflammation, attenuates atherogenesis (Hasturk et al., 2015; Salic et al., 2016), and facilitates myocardial recovery from ischemia in the early stage by suppressing the infiltration of dominant Ly6C^hi^ monocytes/macrophages and the secretion of pro-inflammatory cytokines (Liu et al., 2018b).

Based on these effects of RvE1, in this study, we examined whether RvE1 protects against LPS-induced acute heart injury and further investigated its underlying mechanism.

## Materials and Methods

### Reagents

RvE1 (5S, 12R, 18R-trihydroxy-6Z, 8E, 10E, 14Z, 16E-EPA, purity ≥ 95%, λ_*max*_, 272 nm) was purchased from Cayman Chemical (Ann Arbor, MI, United States). LPS was obtained from Sigma-Aldrich (St. Louis, MO, United States).

### Animals

All experimental procedures complied with the National Institutes of Health (NIH) Guide for the Care and Use of Laboratory Animals and were approved by the Animal Care and Use Committee of Renmin Hospital of Wuhan University (Wuhan, China).

Adult male C57BL/6 mice (aged 6–8 weeks) were purchased from Vital River Laboratory Animal Technology Co. Ltd. (Beijing, China). Mice were maintained in a humidity/temperature-controlled environment (70% relative humidity, 22°C) in a standard laboratory of the Cardiovascular Research Institute of Wuhan University with a 12:12-h light–dark cycle and were supplied with rodent food and water. These animals were acclimatized to the environment for 2 weeks and then randomly assigned into three groups: the control group, LPS exposure group, and LPS + RvE1 group. Mice were treated with LPS (10 mg/kg) *via* once intraperitoneal (i.p.) injection and pretreated i.p. with RvE1 (25 μg/kg) or vehicle (0.9% endotoxin-free saline) 30 min prior to LPS administration. Mice were sacrificed after 6 h of LPS treatment.

### Echocardiography

After 6 h of LPS treatment, mice anesthetized with 1.5–2% isoflurane, were subjected to echocardiographic analysis using a Mylab 30CV ultrasound (Esaote S.P.A., Genoa, Italy) with a 10-MHz linear array ultrasound transducer. The heart rate (HR) and the left ventricular (LV) function, which included the LV ejection fraction (LVEF), fractional shortening (FS), LV end-systolic diameter (LVESD), and LV end-diastolic diameter (LVEDD), were assessed.

### Biochemical Determination

After the echocardiography analysis, animals were maintained under anesthesia, blood samples were taken from each mouse and centrifuged for 15 min at 3,000 × *g*. Then, the serum was used to detect lactate dehydrogenase (LDH) activity and creatine kinase myocardial bound (CK-MB) levels following the manufacturer’s protocols (all from Nanjing Jiancheng Bioengineering Institute, Nanjing, China).

### Histological Analysis

Hearts were isolated and arrested in 10% KCl solution, and spleens were subsequently isolated. Then, after fixation with 4% paraformaldehyde for 5 days, the hearts and spleens were embedded in paraffin and sliced into approximately 5 μm sections. Subsequently, the sections were stained with hematoxylin and eosin (H&E) for histological analysis.

### Immunofluorescence

For immunofluorescence, each heart or spleen section was deparaffinized and blocked with 10% bovine serum albumin. Subsequently, the heart or spleen sections were incubated overnight at 4°C with one of the following primary antibodies: (a) anti-Ly6G antibody, (b) anti-CD68 antibody (R&D Systems), (c) anti-CD206 antibody (R&D Systems), and (d) anti-CD80 antibody (R&D Systems). Then, the sections were washed in phosphate-buffered saline (PBS) and incubated for 1 h at 37°C with secondary antibodies [horseradish peroxidase (HRP) goat anti-rabbit immunoglobulin G (IgG)]. Nuclei were counterstained with 4′,6-diamidino-2-phenylindole (DAPI).

### TdT-Mediated dUTP Nick-End Labeling Assay

TdT-mediated dUTP nick-end labeling (TUNEL) staining was performed as previously described (Ye et al., 2018). Briefly, apoptosis of the left ventricle was assessed with a TUNEL kit (Millipore, United States) following the manufacturer’s instructions. Light microscopy was used to evaluate apoptosis.

### Quantitative Real-Time PCR

Total heart or splenic RNA was extracted with TRIzol reagent (Invitrogen Life Technologies, United States). Oligo(dT) primers and a Transcriptor First Strand cDNA Synthesis kit (Roche, Germany) were used to synthesize cDNA. For the PCR amplification, LightCycler 480 SYBR Green Master Mix (Roche, Germany) was used. The mRNA expression levels of the target genes were normalized to those of glyceraldehyde 3-phosphate dehydrogenase (GAPDH). The quantitative real-time (QRT)-PCR primers are shown in Table 1.

**TABLE 1:** Primers for quantitative real-time PCR.

Gene	Forward primer (5′–3′)	Reverse primer (5′–3′)
IL-1β	GGGCCTCAAAGGAAAGAATC	TACCAGTTGGGGAACTCTGC
IL-6	AGTTGCCTTCTTGGGACTGA	TCCACGATTTCCCAGAGAAC
MCP-1	ACTGAAGCCAGCTCTCTCTTCCTC	TTCCTTCTTGGGGTCAGCACAGAC
BLT1	GGCTGCAAACACTACATCTCC	TCAGGATGCTCCACACTACAA
ChemR23	ATGGAGTACGACGCTTACAACG	GGTGGCGATGACAATCACCA
CD80	GGCCTGAAGAAGCATTAGCTG	GAGGCTTCACCTAGAGAACCG
CD86	GCTTCAGTTACTGTGGCCCT	TGTCAGCGTTACTATCCCGC
CD163	TCCACACGTCCAGAACAGTC	CCTTGGAAACAGAGACAGGC
CD206	CAGGTGTGGGCTCAGGTAGT	TGTGGTGAGCTGAAAGGTGA
CD38	TCTCTAGGAAAGCCCAGATCG	GTCCACACCAGGAGTGAGC
CD36	ATGGGCTGTGATCGGAACTG	TTTGCCACGTCATCTGGGTTT
i-NOS	CGAAACGCTTCACTTCCAA	TGAGCCTATATTGCTGTGGCT
Arg-1	AACACGGCAGTGGCTTTAACC	GGTTTTCATGTGGCGCATTC
COX-1	GATTGTACTCGCACGGGCTAC	GGATAAGGTTGGACCGCACT
COX-2	AACCGCATTGCCTCTGAAT	CATGTTCCAGGAGGATGGAG
5-LOX	TGTTCCCATTGCCATCCAG	CACCTCAGACACCAGATGCG
ALOX-15	AAAGGCACTCTGTTTGAAGCG	CACCAA GTGTCCCCTCAGAAG
Bax	TGAGCGAGTGTCTCCGGCGAAT	GCACTTTAGTGCACAGGGCCTTG
BCL-2	TGGTGGACAACATCGCCCTGTG	GGTCGCATGCTGGGGCCATATA

*ALOX-15, arachidonate 15-lipoxygenase; BLT1, leukotriene B4 (LB4) receptor 1; COX, cyclooxygenase; IL, interleukin; LOX, lipoxygenase; MCP, monocyte chemoattractant protein.*

### Western Blotting

The cardiac tissue protein was extracted, and the protein concentration was assessed, as previously described (Ye et al., 2018). Protein was separated by electrophoresis using Laemmli sodium dodecyl sulfate (SDS)-polyacrylamide gels and then transferred to Immobilon-FL polyvinylidene fluoride (PVDF) membranes (Millipore, United States). Subsequently, after being blocked with 5% non-fat milk for 1 h, the membranes were incubated at 4°C overnight with the following primary antibodies: ChemR23 (Santa Cruz Biotechnology, United States), Bax [Cell Signaling Technology (CST), United States], Bcl-2 (Abcam, United States), c-caspase 3 (CST, United States), phosphorylated/total-p65 (p/T-P65; CST, United States), phosphorylated/total-extracellular signal-regulated kinase (p/T-ERK; CST, United States), phosphorylated/total-c-Jun N-terminal kinase (p/T-JNK; CST, United States), phosphorylated/total-p38 mitogen-activated protein kinase (p/T-P38 MAPK; CST, United States), and GAPDH (CST, United States). Then, the membranes were treated with a second antibody at room temperature for 1 h. Finally, antibody binding was detected with a two-color infrared imaging system (Odyssey, LI-COR Biosciences, Lincoln, United Kingdom). The protein expression intensity was normalized to that of GAPDH.

### Flow Cytometry

Flow cytometry of mice heart tissue was performed as previously described in our study (Wang et al., 2018). Briefly, isolated cell suspensions from hearts were filtered, centrifuged, resuspended, and blocked with a CD16/32 antibody. Samples were then incubated with primary antibodies for 1 h at 4°C in dark. The antibodies include anti-CD45, PE (BD Bioscience), and anti-CD11b+, FITC (BD Bioscience).

### Statistical Analysis

All results are presented as the mean ± standard error of the mean (SEM). Differences between groups were determined by Student’s *t*-test (two groups) or one-way analysis of variance (ANOVA) followed by Dunnett’s test or Tukey’s test (three groups). The significance criterion was set at a *p*-value < 0.05.

## Results

### Lipopolysaccharide Upregulates the Expression of ChemR23 and BLT1 in the Heart

**FIGURE 1:**
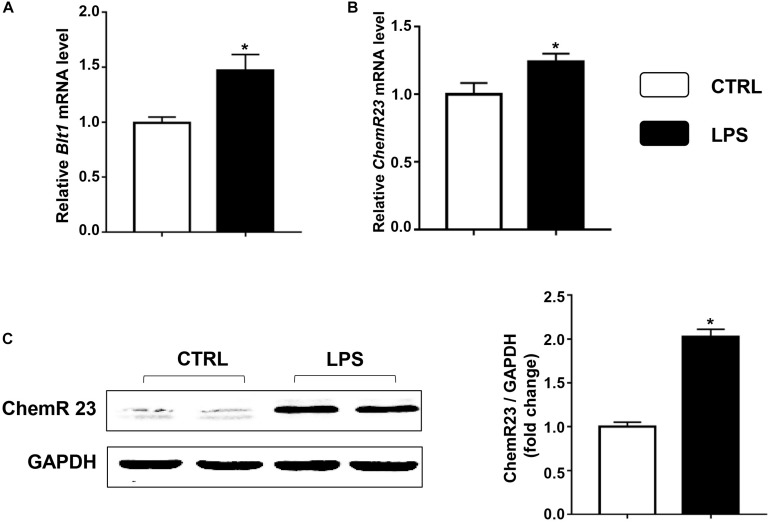
Lipopolysaccharide (LPS) increases the expression of leukotriene B4 (LB4) receptor 1 (BLT1) and ChemR23 in the heart. Relative mRNA levels of BLT1 **(A)** and ChemR23 **(B)** in the control and LPS group (*n* = 5). **(C)** Representative Western blotting bands and quantitative results of protein levels of ChemR23 in the left ventricular (LV) tissue (*n* = 5). Data are presented as the mean ± SEM. *P < 0.05 compared with the control (CTRL) group.

We first examined the expression of ChemR23 and BLT1 in the heart after LPS treatment. According to the QRT-PCR results, LPS treatment significantly increased the cardiac expression of BLT1 (Figure 1A) and ChemR23 (Figure 1B). In addition, Western blotting (WB) results showed that the expression of ChemR23 was increased by the administration of LPS (Figure 1C).

### Resolvin E1 Attenuates Lipopolysaccharide-Induced Myocardial Dysfunction

**FIGURE 2:**
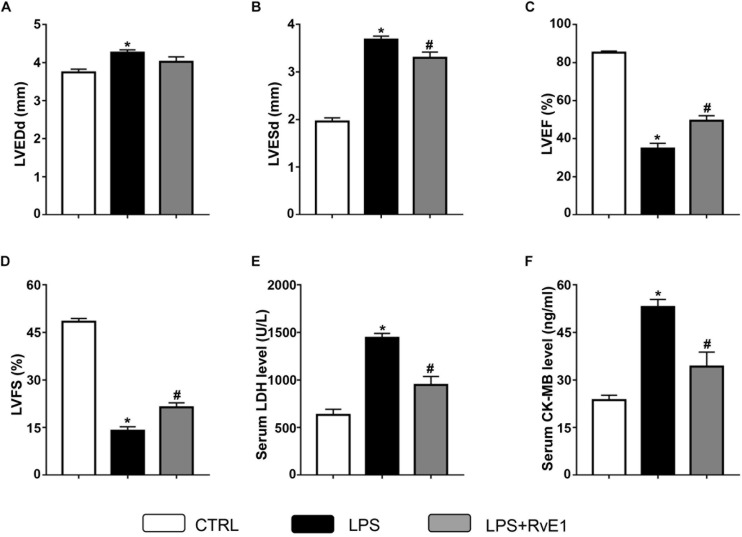
Resolvin E1 ameliorates cardiac function in mice challenged with lipopolysaccharide (LPS). Left ventricular end-diastolic diameter (LVEDD) **(A)**, LV end-systolic diameter (LVESD) **(B)**, LV ejection fraction (LVEF) **(C)**, and LV fractional shortening (LVFS) **(D)** were evaluated by echocardiography in each group (*n* = 6). Serum levels of lactate dehydrogenase (LDH) **(E)** and creatine kinase myocardial bound (CK-MB) **(F)** were measured in each group (*n* = 5). Data are presented as the mean ± SEM. *P < 0.05 compared with the control (CTRL) group, ^#^*P* < 0.05 compared with the LPS group.

Compared to that in the control group, LVEF (Figure 2C) and FS (Figure 2D) in the LPS group were markedly reduced, whereas LVEDD (Figure 2A) and LVESD (Figure 2B) were obviously increased, which suggest that LPS treatment reduces LV function. In contrast, these changes were significantly reversed by RvE1 (25 μg/kg) treatment (Figures 2A–D), which indicates that RvE1 protects LV function in LPS-induced heart injury.

### Resolvin E1 Reduces the Serum Levels of Creatine Kinase Myocardial Bound and Lactate Dehydrogenase in a Lipopolysaccharide-Induced Sepsis Model

To test the effect of RvE1 on LPS-induced myocardial injury, we examined the levels of marker enzymes of myocardial injury in the serum. Compared to those in the control group, the CK-MB and LDH levels in the LPS treatment group were significantly increased (Figures 2E,F). However, pretreatment with RvE1 notably mitigated these changes (Figures 2E,F), which suggests that RvE1 pretreatment alleviates LPS-induced myocardial injury in mice.

### Resolvin E1 Reduces Inflammatory Cytokine Production Induced by Lipopolysaccharide in Cardiac Tissue

**FIGURE 3:**
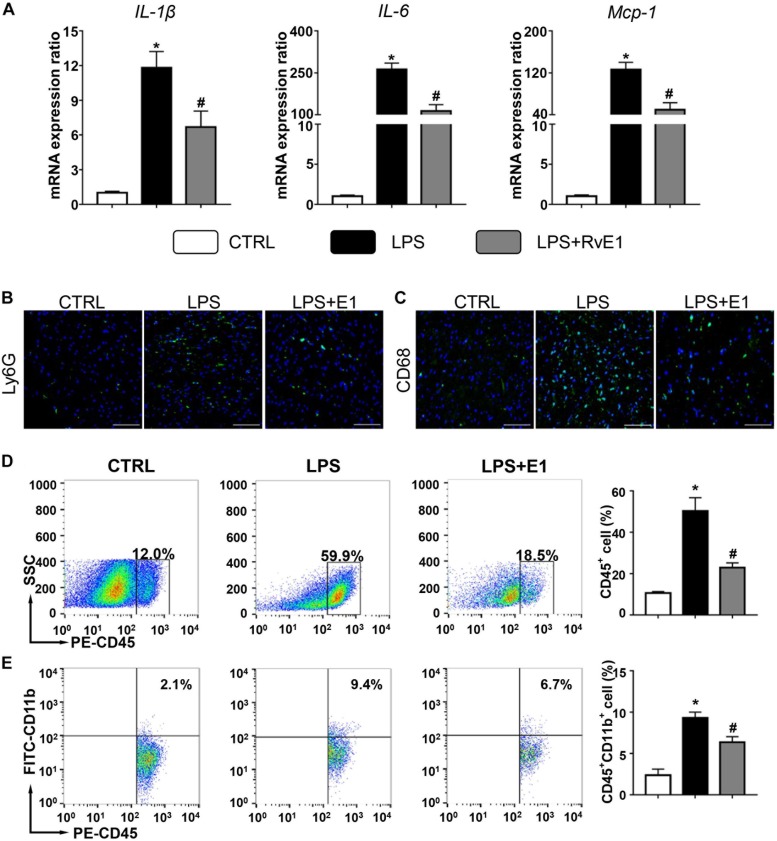
Resolvin E1 inhibits the inflammation response in the heart. **(A)** mRNA levels of pro-inflammatory cytokines [interleukin (IL)-1β, IL-6, and monocyte chemoattractant protein (MCP)-1] were assessed in cardiac tissue (*n* = 5). Infiltration of neutrophils **(B)** and macrophages **(C)** in the heart in different groups (*n* = 4) (scale bar, 50 μm). **(D)** Flow cytometry analysis of CD45+ cells (*n* = 3). **(E)** Flow cytometry analysis of CD45 + CD11b + cells (*n* = 3). Data are presented as the mean ± SEM. *P < 0.05 compared with the control (CTRL) group, ^#^*P* < 0.05 compared with the lipopolysaccharide (LPS) group.

Left ventricular tissue from the three groups was further analyzed for the expression of a panel of inflammatory cytokines, including IL-1β, IL-6, and MCP-1. Compared to those in the control group, the mRNA levels of these inflammatory cytokines were markedly increased after 6 h of LPS exposure. However, the increase in inflammatory cytokine levels was observably inhibited by supplementation with RvE1 (Figure 3A). Similarly, compared to the cecal ligation and puncture (CLP) group, the mRNA levels of IL-1β and IL-6 were significantly reduced by treatment of RvE1 (Supplementary Figures 1A,B).

### Resolvin E1 Reduces the Infiltration of Inflammatory Cells in the Heart

We also evaluated the infiltration of inflammatory cells in the heart by immunofluorescence. Compared to that in the control group, the infiltration of CD68+ macrophages and Ly6G+ neutrophils in the heart in the LPS group were significantly increased (Figures 3B,C). Interestingly, these changes were obviously mitigated by RvE1 treatment (Figures 3B,C). Similarly, these trends were also observed in CLP mouse models (Supplementary Figures 1E,F). Flow cytometry results also showed that LPS increased the infiltration of CD45+ cells and CD45+ CD11b+ cells in the heart, which was reversed by pretreatment of RvE1 (Figures 3D,E). These results indicate that RvE1 reduces the infiltration of inflammatory cells in the heart.

### Resolvin E1 Mediates Macrophage Polarization in the Heart

**FIGURE 4:**
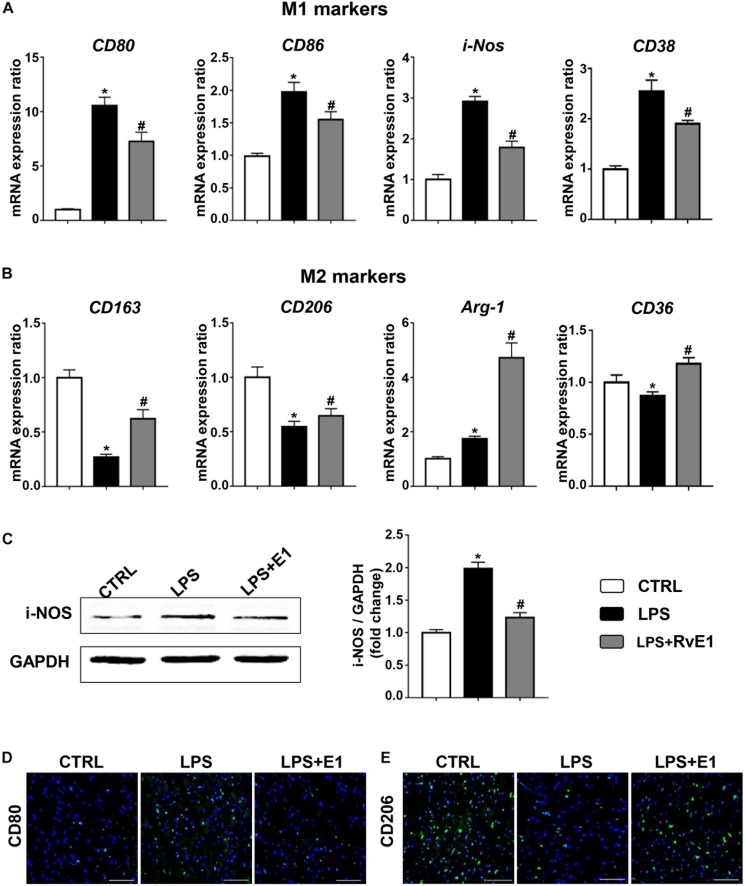
Resolvin E1 protects mice against lipopolysaccharide (LPS)-induced heart injury through the reprogramming of macrophage polarization in the heart. **(A)** mRNA levels of M1 markers, including CD80, CD86, CD38, and i-NOS, in the left ventricular (LV) tissue of each group (*n* = 5). **(B)** mRNA levels of M2 markers, including CD163, CD206, CD36, and Arg-1, in the LV tissue of each group (*n* = 5). **(C)** i-NOS was assessed by Western blotting in the LV tissue of each group (*n* = 4). **(D)** CD80 was assessed by immunofluorescence in the LV tissue of each group (*n* = 4) (scale bar, 50 μm). **(E)** CD206 was assessed by immunofluorescence in the LV tissue of each group (*n* = 4) (scale bar, 50 μm). Data are presented as the mean ± SEM. *P < 0.05 compared with the control (CTRL) group, ^#^*P* < 0.05 compared with the LPS group.

To analyze the effect of RvE1 on macrophage differentiation in LPS-induced myocardial injury, we first detected the expression of surface markers or soluble regulators of macrophages in the heart using QRT-PCR. The mRNA levels of M1 markers, including CD80, CD86, CD38, and i-NOS, were observably increased in the LPS group, but pretreatment with RvE1 significantly inhibited this increase (Figure 4A). Conversely, treatment with LPS reduced the expression of M2 markers (CD163, CD206, and CD36), and RvE1 treatment significantly reversed this trend (Figure 4B). In addition, compared to the CLP group, RvE1 also reduced the mRNA level of CD38 and increased the level of Arg-1 (Supplementary Figures 1C,D). Then, we also evaluated the expression levels of i-NOS by WB; the results revealed that the levels of i-NOS were lower in the LPS + RvE1 group than those in the LPS group (Figure 4C). In addition, immunofluorescence staining showed similar results: lower CD80 expression (Figure 4D) and higher CD206 expression (Figure 4E) were observed in the LPS + RvE1 group than the LPS group. These results suggest that in cardiac tissue, RvE1 may inhibit LPS or CLP-induced MI polarization and promote macrophage polarization toward the M2 phenotype, which suppresses inflammation and promotes the resolution of inflammation.

### Resolvin E1 Reduces the Infiltration of Inflammatory Cells and Mediates Macrophage Polarization in the Spleen

**FIGURE 5:**
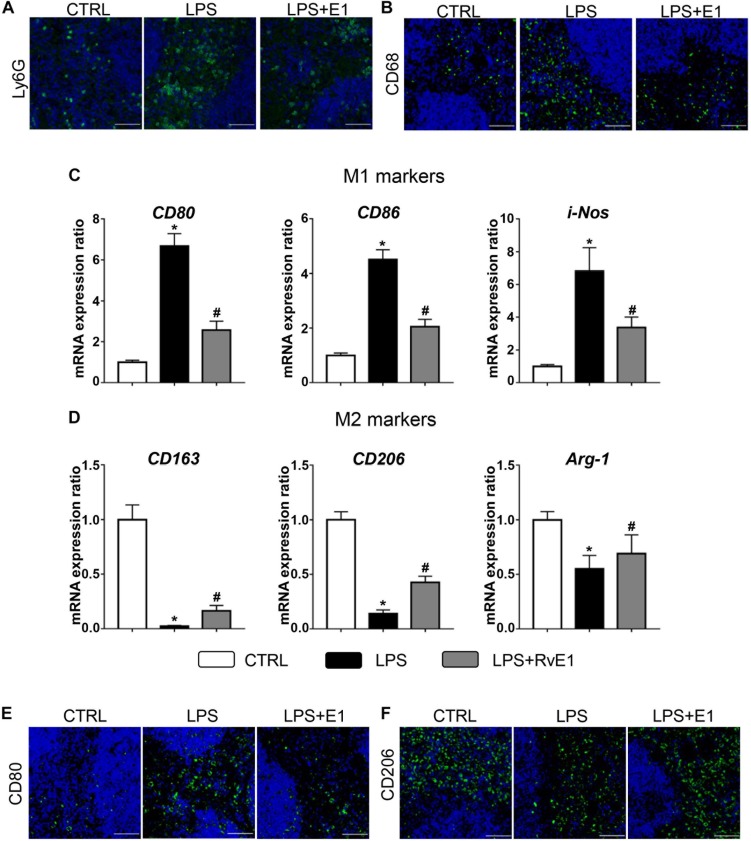
Resolvin E1 reduces the infiltration of inflammatory cells and mediates macrophage polarization in the spleen. **(A,B)** Infiltration of neutrophils and macrophages in the spleen in different groups (*n* = 4) (scale bar, 50 μm). **(C)** mRNA levels of M1 markers, including CD80, CD86, and i-NOS, in the spleen of each group (*n* = 5). **(D)** mRNA levels of M2 markers, including CD163, CD206, and Arg-1, in the spleen of each group (*n* = 5). **(E)** CD80 was assessed by immunofluorescence in the spleen of each group (*n* = 4) (scale bar, 50 μm). **(F)** CD206 was assessed by immunofluorescence in the spleen of each group (*n* = 4) (scale bar, 50 μm). Data are presented as the mean ± SEM. *P < 0.05 compared with the control (CTRL) group, ^#^*P* < 0.05 compared with the lipopolysaccharide (LPS) group.

Immunofluorescence results showed that RvE1 reduced the infiltration of CD68+ macrophages and Ly6G+ neutrophils in the spleen challenged with LPS (Figures 5A,B). We also evaluated the effect of RvE1 on macrophage polarization in the spleen. Treatment with LPS increased the mRNA levels of M1 markers (CD80, CD86, and i-NOS) and reduced the levels of M2 markers (CD163, CD206, and Arg-1). However, pretreatment with RvE1 significantly diminished these trends (Figures 5C,D). Similarly, immunofluorescence staining revealed that the expression levels of CD80 (Figure 5E) were notably reduced, and the expression levels of CD206 (Figure 5F) were significantly increased in the LPS + RvE1 group compared to those in the LPS group. These results reveal that RvE1 may also suppress the inflammation of the spleen by promoting macrophage M2 polarization and inhibiting M1 polarization, ultimately accelerating the resolution of systemic inflammation induced by LPS.

### Resolvin E1 Mediates the Expression of Cyclooxygenase and Lipoxygenase in the Myocardial Tissue of Lipopolysaccharide-Treated Mice

**FIGURE 6:**
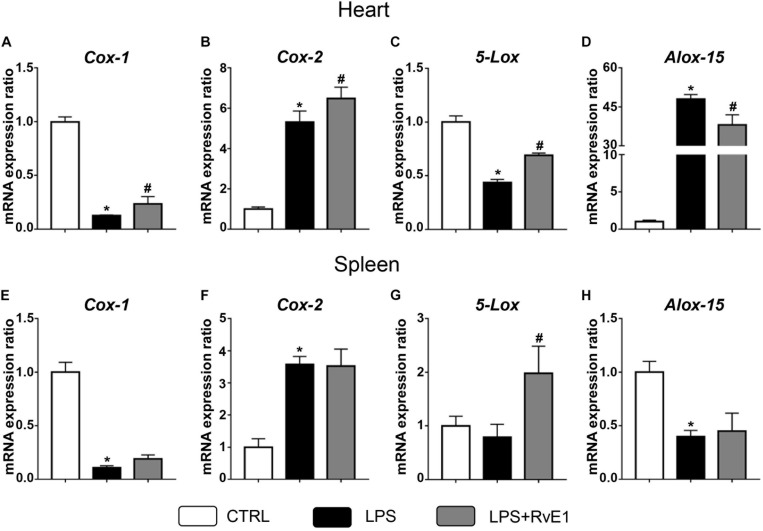
Resolvin E1 mediates the expression of cyclooxygenase (COX) and lipoxygenase (LOX) in the myocardial tissue of lipopolysaccharide (LPS)-treated mice. **(A–D)** COX-1, COX-2, 5-LOX, and arachidonate 15-lipoxygenase (ALOX-15) in the left ventricular (LV) tissue were evaluated by QRT-PCR in each group (*n* = 5). **(E–H)** COX-1, COX-2, 5-LOX, and ALOX-15 in the spleen were evaluated by QRT-PCR in each group (*n* = 5). Data are presented as the mean ± SEM. *P < 0.05 compared with the control (CTRL) group, ^#^*P* < 0.05 compared with the LPS group.

Previous studies have suggested that COX and LOX may play a critical role in the formation of lipid mediators and the regulation of inflammation (Kain et al., 2014). In the present study, QRT-PCR was used to evaluate the expression of these immune-sensitive enzymes. We found that LPS treatment significantly reduced the mRNA levels of COX-1 and 5-LOX in the heart, and this effect was reversed by RvE1 (Figures 6A,C). On the contrary, arachidonate 15-LOX (ALOX-15) levels were increased in the myocardial tissue of LPS-treated mice, and pretreatment with RvE1 obviously mitigated the trend compared to LPS treatment alone (Figure 6D). In addition, the mRNA levels of COX-2 were significantly increased in the LPS group and further increased in the LPS + RvE1 group (Figure 6B). These results suggest that RvE1 mitigates the inflammation of cardiac tissue possibly associated with the modulation of lipid mediator-related enzymes. However, the association between RvE1 and lipid mediator-related enzymes in spleen remains to be further studied (Figures 6E–H).

### Resolvin E1 Reduced Lipopolysaccharide-Induced Myocardial Apoptosis *in vivo* and *in vitro*

**FIGURE 7:**
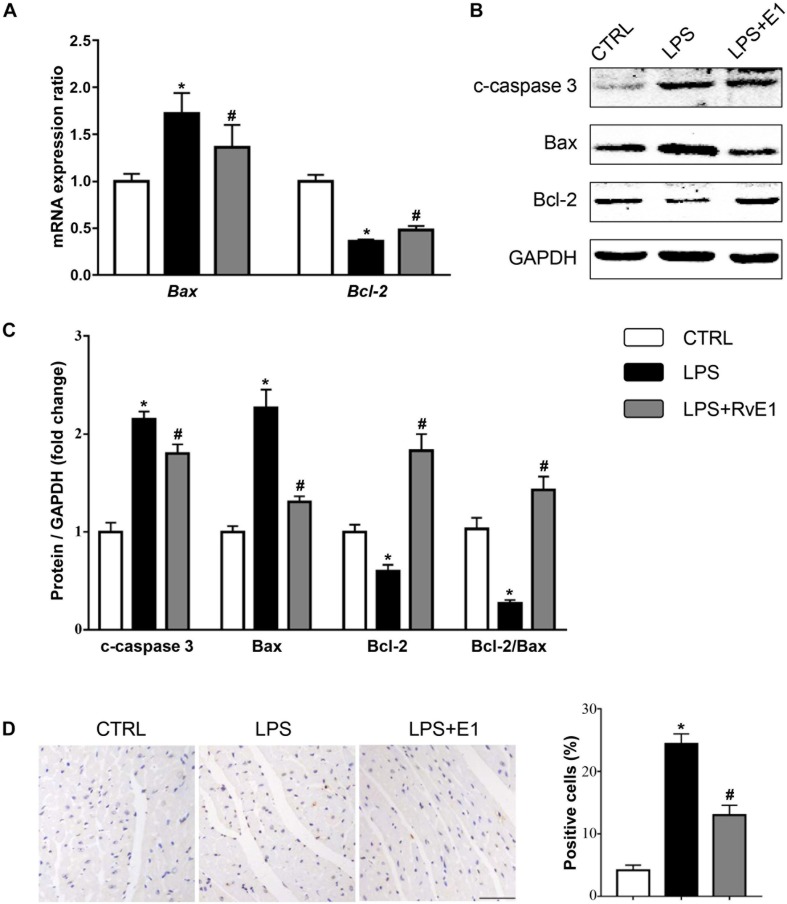
Resolvin E1 attenuates lipopolysaccharide (LPS)-induced cardiomyocyte apoptosis. **(A)** mRNA levels of Bax and Bcl-2 in each group (*n* = 5). **(B,C)** Representative Western blotting bands **(B)** and quantitative results **(C)** of c-caspase-3, Bax, and Bcl-2 in each group (*n* = 5). **(D)** Representative images of TdT-mediated dUTP nick-end labeling (TUNEL) staining and the quantitative results in each group (*n* = 4) (scale bar, 50 μm). Data are presented as the mean ± SEM. *P < 0.05 compared with the control (CTRL) group, ^#^*P* < 0.05 compared with the LPS group.

To assess whether RvE1 prevents LPS-induced cardiomyocyte apoptosis, we first detected the activation of apoptosis-related signaling pathways. The QRT-PCR results revealed that the mRNA levels of Bax were higher and the levels of Bcl-2 were lower in the LPS group than those in the control group (Figure 7A). However, the expression of Bax was reduced and the expression of Bcl-2 was increased in the RvE1-pretreatment group compared to those in the LPS group (Figure 7A). Similarly, WB results also suggested that Bax and c-caspase 3 levels were lower and the Bcl-2 level was higher in the LPS + RvE1 group than those in the LPS group (Figures 7B,C). In addition, after treatment with LPS for 6 h, an increase in the number of TUNEL-positive cells was observed in mice, and pretreatment with RvE1 alleviated this trend (Figure 7D). *In vitro*, WB results showed similar results (Supplementary Figures 2A–C).

### Resolvin E1 Inhibits the Activation of the Mitogen-Activated Protein Kinase and Nuclear Factor-κB Signaling Pathways in the Myocardial Tissue of Lipopolysaccharide-Treated Mice

**FIGURE 8:**
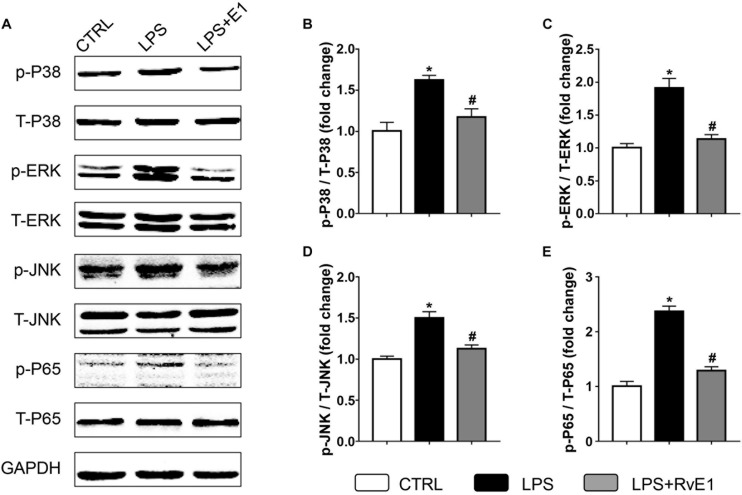
Resolvin E1 inhibits the activation of the mitogen-activated protein kinase (MAPK) and nuclear factor (NF)-κB signaling pathways in the myocardial tissue of lipopolysaccharide (LPS)-treated mice. **(A)** Representative Western blotting bands of p/T-p38, p/T-ERK, p/T-JNK, and p/T-p65 in each group. Quantitative results of p-P38 **(B)**, p-ERK **(C)**, p-JNK **(D)**, and p-P65 **(E)** in each group (*n* = 5). Data are presented as the mean ± SEM. *P < 0.05 compared with the control (CTRL) group, ^#^*P* < 0.05 compared with the LPS group.

We further examined the activation of MAPK and NF-κB signaling pathways, which play a key role in regulating the production of inflammatory mediators, in cardiac tissue. Our results revealed that phosphorylation of P38, c-Jun N-terminal kinase (JNK), extracellular signal-regulated kinase (ERK), and P65 were increased in LV myocardial tissue from the LPS group (Figures 8A–E). However, levels of these LPS-activated signaling molecules were markedly reduced by RvE1 treatment (Figures 8A–E).

## Discussion

In the present study, we reveal that RvE1 attenuates LPS-induced cardiomyocyte injury, as evidenced by the improvement in cardiac function, decrease in the expression of myocardial damage markers, and reduction in the levels of pro-inflammatory cytokines. We also observed that the expression of the RvE1 receptors ChemR23 and BLT1 was increased after treatment with LPS, indicating that RvE1 might exert a protective effect by interacting with these receptors. In addition, RvE1 treatment inhibited the infiltration of inflammatory cells, regulated M1/M2 macrophage polarization in the heart and spleen, and modulated the expression of COX and LOX in the heart, which correlated with the resolution of inflammation. In addition, RvE1 also inhibited the MAPK and NF-κB inflammatory signaling pathways and mitigated the apoptosis of myocardial cells. In summary, these findings indicate that RvE1 might be a potent lipid metabolite that exerts protective effects against sepsis-induced cardiac injury by inhibiting local and systemic inflammatory responses, modulating macrophages polarization and reducing the rate of myocardial apoptosis.

Sepsis-induced cardiomyopathy is associated with high morbidity and mortality rates in critically ill patients (Romero-Bermejo et al., 2011; Deutschman and Tracey, 2014). LPS is frequently used to induce *SIC* in mice. It has been proposed that experimental septic myocardial injury was mainly evidenced by myocardial dysfunction and elevation of myocardial injury markers, including LDH, CK, and CK-MB (Chen et al., 2017, 2018). RvE1, an omega-3 polyunsaturated fatty acid (PUFA)-derived metabolite, exhibits anti-inflammatory or proresolving activity in multiple animal models of inflammatory diseases, such as periodontal inflammation, psoriatic dermatitis, and acute allergic asthma (Flesher et al., 2014; Balta et al., 2017; Sawada et al., 2018). In addition, previous research has indicated that RvE1 attenuates LPS-induced inflammation *in vitro* (Baker et al., 2018). As previously mentioned, RvE1 also plays a protective role in multiple CVDs [coronary artery disease (CAD)], including atherosclerosis, MI, and reperfusion injury (Keyes et al., 2010; Hasturk et al., 2015; Liu et al., 2018b). Therefore, it is highly necessary to clarify the functional roles of RvE1 in *SIC*. Interestingly, we found that LPS treatment increased serum LDH and CK-MB levels, and this increase was reversed by pretreatment with RvE1. In addition, RvE1 improved LVEF and FS in hearts challenged with LPS. These results suggest that RvE1 plays a protective role in septic heart injury.

Inflammatory cells and inflammatory cytokines play an important role in the inflammatory response to pathologic stimuli. Myocardial infiltration by immune cells, especially neutrophils and macrophages, contributes to sepsis-induced cardiac dysfunction, which is attenuated by the inhibition of the influx of these cells (Chen J. et al., 2016; Zheng et al., 2017). The levels of pro-inflammatory cytokines, including tumor necrosis factor (TNF)-α, IL-1β, IL-6, and MCP-1, have been observed to increase in the myocardium in response to sepsis (Chen J. et al., 2016; Huang et al., 2018). These cytokines are considered to be myocardial depression factors and exert an injurious effect on cardiomyocytes. Additionally, the neutralization of IL-6 and IL-1β contributes to the inhibition of the inflammatory response (Eger et al., 2018). In the present study, LPS treatment increased the mRNA levels of IL-1β, IL-6, and MCP-1 in cardiac tissue, and this increase was reduced by RvE1 pretreatment. Similarly, RvE1 reduced the sepsis-induced infiltration of neutrophils and macrophages in myocardial tissues. Consistent with this research, a previous study suggested that the anti-inflammatory mediator RvE1 reduces LPS-induced inflammation *in vitro* (Baker et al., 2018). Therefore, RvE1 may protect mouse hearts from septic injury by suppressing inflammation. In addition, the overexpression of the RvE1 receptors BLT1 and ChemR23 enhances the anti-inflammatory effect of RvE1 (Arita et al., 2007; Gao et al., 2013; Herrera et al., 2015; Sima et al., 2017). In this study, LPS treatment increased the expression of BLT1 and ChemR23 in the myocardium, which contributed to the protective effect of RvE1 against *SIC*.

The production of pro-inflammatory mediators is closely regulated by the activation of multiple signaling pathways, including MAPKs (ERK, JNK, and p38) and NF-κB. In the heart, LPS promotes the phosphorylation of ERK, JNK, p38, and p65, which enhances the production of pro-inflammatory mediators (Chen R.C. et al., 2016; Zhao et al., 2016). *In vitro*, RvE1 suppresses the production of cytokines in pulmonary macrophages by reducing the nuclear translocation of NF-κB p65 (Flesher et al., 2014). However, whether RvE1 prevents the phosphorylation of MAPK and p65 in *SIC* is still unknown. Our results revealed that the expression levels of p-ERK, p-JNK, p-P38, and p-P65 were lower in the RvE1 + LPS group than those in the LPS group, which occurred in parallel with the reduction in the levels of pro-inflammatory mediators. Therefore, the protective effect of RvE1 in LPS-induced heart injury may be closely associated with the inhibition of MAPK and NF-κB signaling pathways.

Modulation of the macrophage phenotype plays a crucial role in inflammatory processes and might be a novel strategy for the treatment of sepsis (Mahajan et al., 2015; Guo et al., 2018). LPS induces macrophage polarization to the M1 phenotype, which is associated with pro-inflammatory mediator production and heart injury. In contrast, M2 macrophages secrete increased amounts of anti-inflammatory cytokines and protect the heart against LPS-induced injury (Dai et al., 2015). Previous studies have suggested that protectin D1 regulates macrophage function and that RvD1 stimulates the M2 macrophage phenotype, which is associated with the resolution of lung inflammation and adipose tissue inflammation (Titos et al., 2011; Serhan et al., 2014). RvE1 was found to promote macrophage polarization toward an M2-like phenotype, and these macrophages exerted a protective effect against vascular inflammation induced by mechanical injury (Liu et al., 2018a). Interestingly, our results revealed that RvE1 promotes macrophage polarization toward the M2-like phenotype (CD163, CD206, and Arg-1) and inhibited LPS-induced M1 macrophage polarization (CD80, CD86, and i-NOS). Therefore, RvE1 might reduce the LPS-induced inflammation response by regulating the reprogramming of macrophages. In addition, recent studies have demonstrated that CD163 plays a potentially proresolution role in models of resolving inflammation possibly by mediating IL-10 release and heme oxygenase-1 synthesis (Gilroy and De Maeyer, 2015). In this study, the expression of CD163 was increased in the RvE1 group compared to that in the LPS group, which indicates that RvE1 might play an important role in the resolution phase of LPS-induced heart injury.

Immunometabolism, primarily driven by COX and LOX, is closely associated with inflammation, especially following MI (Kain et al., 2014). Previous research has suggested that COX-1 might play a significant anti-inflammatory role in diabetes-impaired wound tissue by driving PG synthesis, which contributes to the tightly controlled resolution of inflammation (Goren et al., 2006). In our study, RvE1 treatment significantly increased the expression of COX-1, indicating that RvE1 contributes to the resolution of inflammation in *SIC*. The inhibition of COX-2 has a protective effect in many inflammatory diseases, whereas COX-2 still exerts biological activities to resolve inflammation in the resolution phase. The RvD1-induced activation of COX-2 expedites the resolution of inflammation and has therapeutic potential for the management of acute respiratory distress syndrome (Gao et al., 2017). Consistent with the results of previous studies, we found that the expression of COX-2 was increased after 6 h of LPS administration and was further increased by RvE1 treatment, suggesting that RvE1 improves the resolution of inflammation by enhancing the activation of COX-2 in the resolution phase. 5-LOX contributed to the synthesis of resolvins. Previous studies revealed that omega-3-enriched lipid emulsions enhanced macrophage efferocytosis and phagocytosis through 5-LOX (Korner et al., 2018). ALOX-15 is an enzyme that can produce 12-hydroxy-eicosatetraenoic acid (HETE) from arachidonic acid. The inhibition of ALOX-15 might inhibit inflammation and reduce vascular permeability by eliminating 12-HETE production in acute lung injury (Zarbock et al., 2009). Interestingly, RvE1 also reduced the expression of ALOX-15 and increased the expression of 5-LOX, which was consistent with the improvement in cardiac function. Therefore, RvE1 might be tightly associated with the modulation of lipid mediator-related enzymes in *SIC*.

Myocardial apoptosis is also a crucial process during *SIC*, and inhibition of apoptosis has been found to be protective (Sharma, 2007). RvE1 administration protected cardiomyocytes against apoptosis and improved cardiac function by reducing the secretion of pro-inflammatory cytokines in the peri-infarct zones (Liu et al., 2018b). Therefore, we used TUNEL staining, QRT-PCR, and WB to detect cardiomyocyte apoptosis in *SIC*. Our results showed that the number of TUNEL-positive nuclei and the expression of Bax and c-caspase 3 were reduced by RvE1 treatment, whereas the expression of Bcl-2 was increased. These results demonstrate that RvE1 significantly protects cardiomyocytes against sepsis-induced apoptosis.

The spleen is an important peripheral immune organ that is a critical reservoir of immune phagocytes, including neutrophils and monocytes/macrophages, and has been reported to play a crucial role in the clearance of pathogens in sepsis (Jahng et al., 2016; Kapellos et al., 2017). Previous studies have reported that the reduction in the number of neutrophils in the spleen is closely associated with the improvement of cardiac function and inhibition of the inflammatory response (Kain et al., 2015). In addition, M2 macrophage polarization in the spleen has a significantly beneficial effect on inflammation post-MI and sepsis (Kain et al., 2015; Taratummarat et al., 2018). Therefore, the crosstalk between the heart and spleen may affect the regulation of inflammation. In this study, the LPS-induced increase in neutrophil and macrophage densities in the spleen was attenuated by RvE1; this effect was also observed in the heart. In addition, the inhibition of M1 polarization and the induction of M2 polarization have been observed to contribute to the improvement of sepsis after RvE1 administration.

## Conclusion

In conclusion, our results indicate that RvE1 protects the heart from sepsis-induced heart injury. The mechanism is possibly associated with the inhibition of MAPK and NF-κB inflammatory signaling pathways, modulation of macrophage polarization, and reduction in myocardial apoptosis. These findings suggest that RvE1 might be a high potential lipid mediator for the treatment of *SIC*.

## Data Availability Statement

All datasets generated for this study are included in the article/Supplementary Material.

## Ethics Statement

The animal study was reviewed and approved by the Animal Care and Use Committee of Renmin Hospital of Wuhan University (Wuhan, China).

## Author Contributions

JZ and MW contributed to the experimental design and wrote the manuscript. JY, ZW, and YX contributed to the acquisition and analysis of the data. DY, YX, JL, MZ, and JW reviewed the manuscript.

## Conflict of Interest

The authors declare that the research was conducted in the absence of any commercial or financial relationships that could be construed as a potential conflict of interest.

## Abbreviations

BLT1: leukotriene B4 (LB4) receptor 1
CAD: cardiovascular disease
COX: cyclooxygenase
EPA: eicosapentaenoic acid
HEPE: hydroxy-5Z,8Z,11Z,14Z,16E-eicosapentaenoic acid
HETE: hydroxy-eicosatetraenoic acid
LOX: lipoxygenase
LPS: lipopolysaccharide
MI: myocardial infarction
PG: prostaglandin
PUFA: polyunsaturated fatty acid
*SIC*: sepsis-induced cardiomyopathy.
