# Supplementary material for: The Anti-inflammatory Mediator Resolvin E1 Protects Mice Against Lipopolysaccharide-Induced Heart Injury
Source: Front Pharmacol. 2020 Mar 18;11:203. doi: 10.3389/fphar.2020.00203 (PMC7094758; doi:10.3389/fphar.2020.00203)
Supplement: Supplementary file 2 [file Data_Sheet_2.pdf]

SUPPLEMENTARY MATERIAL

The anti-inflammatory mediator resolvin E1 protects mice against lipopolysaccharide-induced heart injury

Jishou Zhang[1,2,3,†], Menglong Wang[1,2,3,†], Jing Ye[1,2,3,†], Jianfang Liu[1,2,3], Yao Xu[1,2,3], Zhen Wang[1,2,3] Di Ye[1,2,3], Mengmeng Zhao[1,2,3], and Jun Wan[1,2,3,*]

[1] Department of Cardiology, Renmin Hospital of Wuhan University, Wuhan, China

[2] Cardiovascular Research Institute, Wuhan University, Wuhan, China

[3] Hubei Key Laboratory of Cardiology, Wuhan, China

Supplemental Methods

Animal Model:

The mice were randomly assigned into three groups: the control group, cecal ligation and puncture (CLP) group, and CLP + RvE1 group. CLP was performed as previously described[1]. Briefly, the mice were anesthetized by isoflurane inhalation and were performed skin preparation. Then, a standard-practice midline incision was made in the abdomen of mice and the cecum were exposed fully. The cecum was ligated 1 cm from the cecum tail and perforated with a 20 G needle, while little feces were squeezed. Finally, we repositioned the cecum and sutured the abdominal incision. The CLP + RvE1 group were treated with RvE1 (25ug/kg) at 1 hour after CLP surgery. After 24 hours, mice were sacrificed for further study.

Immunofluorescence, QT-PCR and western blotting

Immunofluorescence and QT-PCR were performed as described in the manuscript.

Cell selection and culture

RAW264.7 cell macrophages were conventionally cultured in 1640 medium, which contained 10% fetal bovine serum. Primary cardiomyocytes were isolated from neonatal mouse hearts, as described previously[2]. RAW264.7 cell was incubated in a new 1640 medium and supplemented with vehicle, LPS (50ng/ml) and LPS+ RvE1 (100nM). After 12 hours, the cellular supernatant was used to culture cardiomyocytes, which were recruited in 24 hours later. Finally, western blotting was used to detected the apoptosis of cardiomyocytes.

Statistical Analysis

All results are presented as the mean ±standard error of the mean (SEM). Differences between groups were determined by Student's t test (2 groups) or one-way analysis of variance (ANOVA) followed by Dunnett's test or Tukey's test (3 groups). The significance criterion was set at a p value < 0.05.

Supplementary Figures and Figure Legends

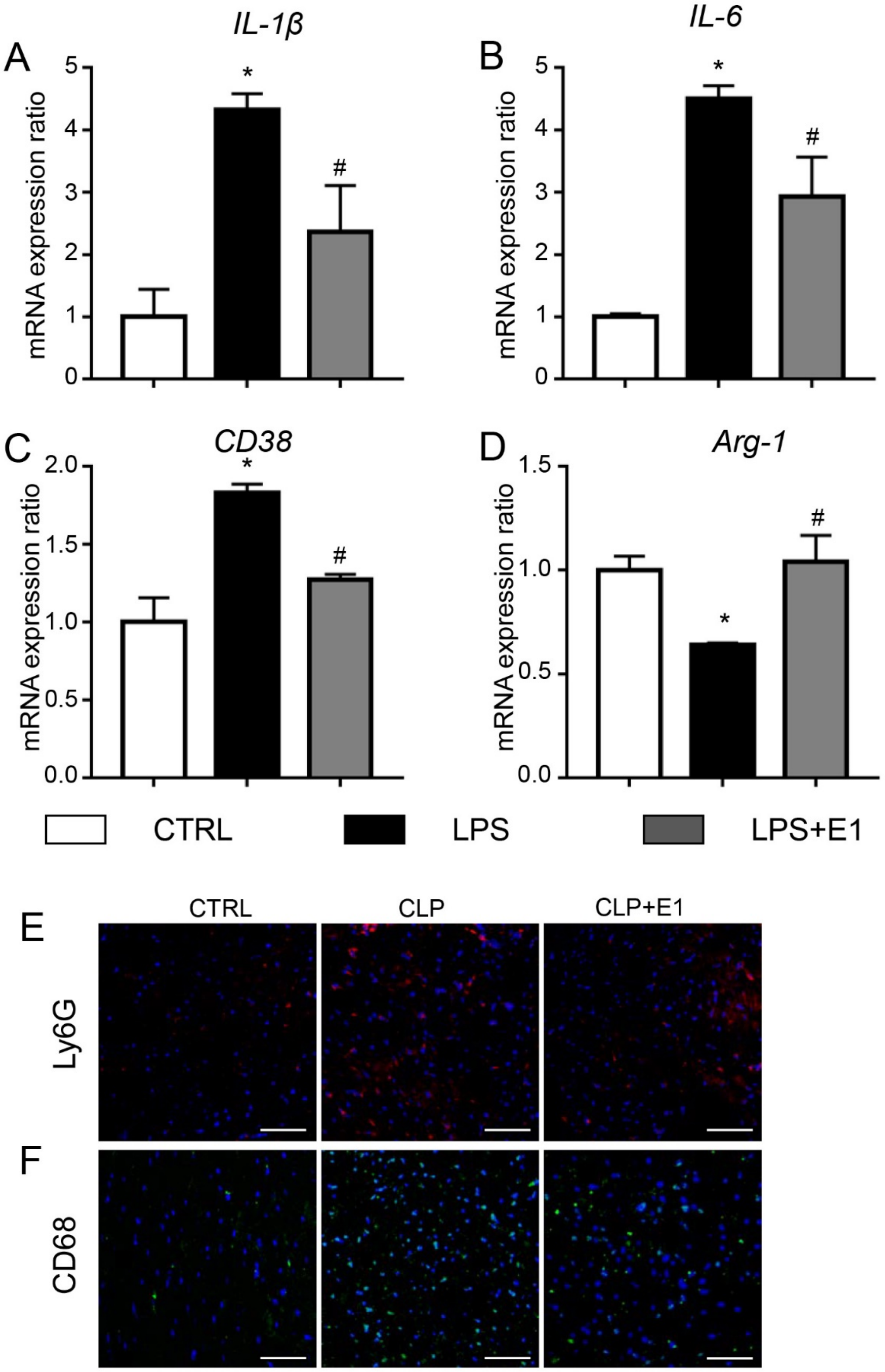

Supplementary Figure 1 RvE1 inhibited inflammation and mediated macrophages polarization in heart from CLP mice. A. mRNA levels of IL-1β, n=4; B. mRNA levels of IL-6, n=4; C. mRNA levels of CD38, n=4; D. mRNA levels of Arg-1, n=4; E.

Immunofluorescence staining of Ly6G (red), bar=50μm， n=4; F. Immunofluorescence staining of CD68 (green), bar=50μm， n=4.

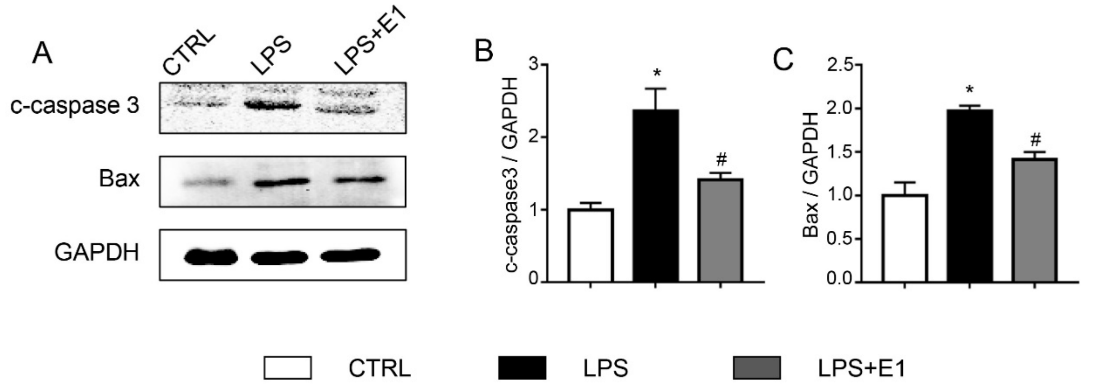

Supplementary Figure 2 RvE1 attenuated the apoptosis of cardiomyocytes in vitro. A. Western blotting stain of c-caspase 3, bax and GAPDH. B-C. The quantitative results of c-caspase3 and bax, n=3.

Supplementary References:

1. Sun S, Wang J, Wang J, Wang F, Yao S, Xia H. Maresin 1 Mitigates Sepsis-Associated Acute Kidney Injury in Mice via Inhibition of the NF-kappaB/STAT3/MAPK Pathways. *FRONT PHARMACOL*. 2019;10:1323.

2. Wang Y, Li Z, Zhang Y, Yang W, Sun J, Shan L, Li W. Targeting Pin1 Protects Mouse Cardiomyocytes from High-Dose Alcohol-Induced Apoptosis. *OXID MED CELL LONGEV*. 2016;2016:4528906.
